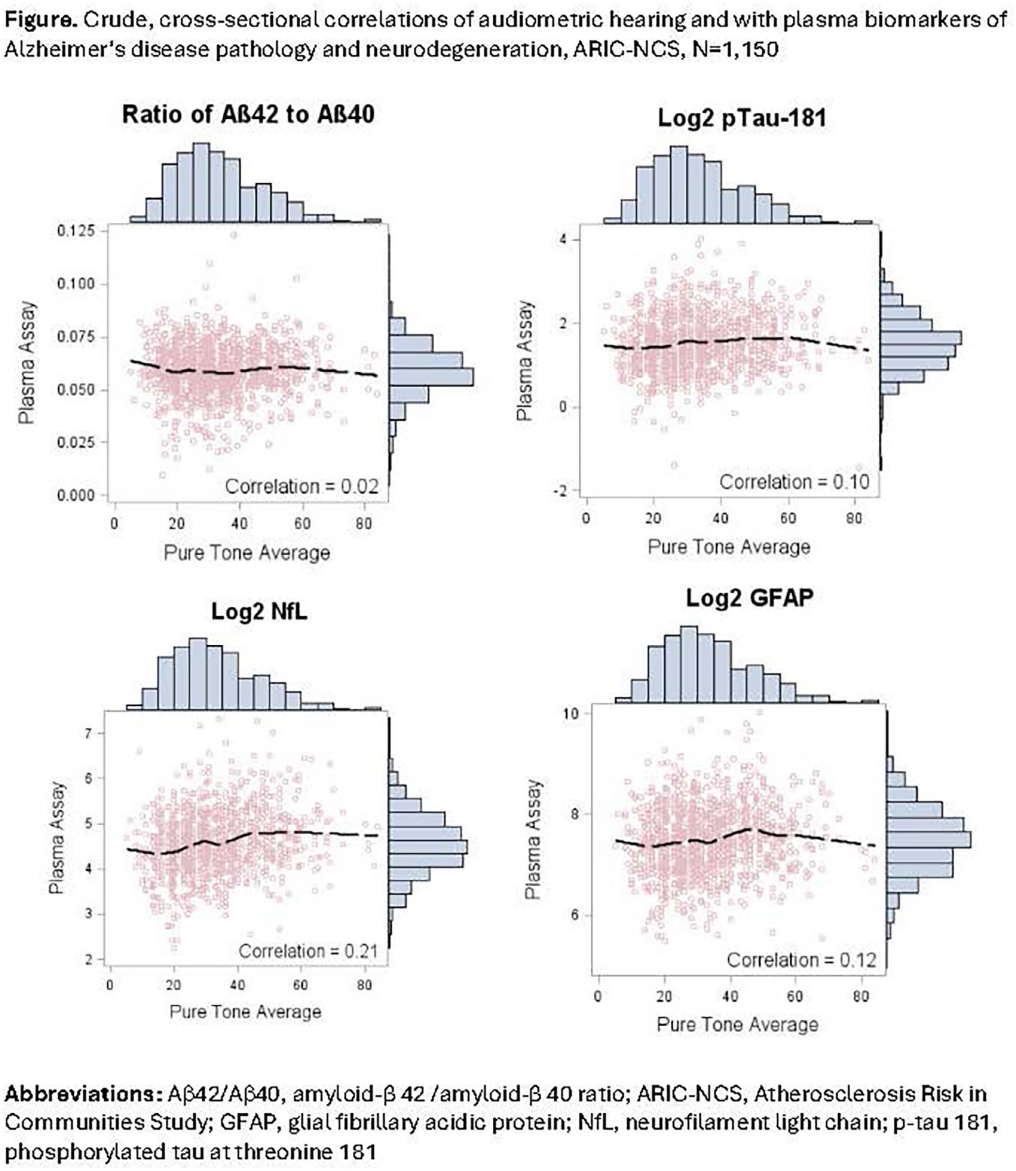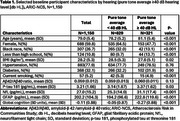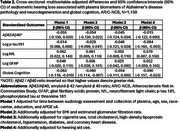# Audiometric hearing and plasma biomarkers of Alzheimer's disease pathology and neurodegeneration in the Atherosclerosis Risk in Communities Neurocognitive Study

**DOI:** 10.1002/alz70860_106812

**Published:** 2025-12-23

**Authors:** Jennifer A. Deal, James Russell Pike, Vidyulata Kamath, Priya Palta, Bharat Thyagarajan, Frank R Lin, Josef Coresh, Nicholas S Reed

**Affiliations:** ^1^ Johns Hopkins Bloomberg School of Public Health, Baltimore, MD, USA; ^2^ Departments of Population Health and Medicine, New York University Grossman School of Medicine, New York, NY, USA; ^3^ Johns Hopkins University School of Medicine, Baltimore, MD, USA; ^4^ University of North Carolina at Chapel Hill, Chapel Hill, NC, USA; ^5^ University of Minnesota, Minneapolis, MN, USA; ^6^ New York University, New York, NY, USA

## Abstract

**Background:**

Hearing loss is an established risk factor for Alzheimer's disease and related dementias (ADRD) but the mechanism is unknown. We investigated the cross‐sectional association of hearing loss with plasma AD/ADRD biomarkers, hypothesizing that hearing loss is associated with biomarkers reflective of broader neurodegeneration (e.g., neurofilament light chain [NfL]) but not with AD‐specific markers (e.g., amyloid‐b, phosphorylated tau).

**Methods:**

We used data from the observational cohort, the Atherosclerosis Risk in Communities Neurocognitive Study (ARIC‐NCS). Pure tone air conduction hearing thresholds (frequencies 0.5‐4 kHz) were obtained at Visit 6 (2016‐17) and averaged, with better‐ear hearing loss modeled continuously. The Quanterix SiMoA platform measured ADRD plasma biomarkers from stored specimens at the nearest visit – Visit 5 (2011‐13), Visit 6 (2016‐17) or Visit 7 (2018‐19) – including amyloid‐β 42 /amyloid‐β 40 ratio (Ab42/40), phosphorylated tau at threonine 181 (*p*‐tau 181), NfL, and glial fibrillary acidic protein (GFAP). Multivariable‐adjusted linear regression was used to estimate differences in biomarker levels, adjusting for time between audiology assessment and plasma collection, age, sex, race, center, education, *APOE* e4 genotype, BMI, estimated glomerular filtration rate, cigarette use, total cholesterol, high‐density lipoprotein cholesterol, hypertension, diabetes, coronary heart disease and hearing aid use. Global cognition (a composite cognitive score created from 10 cognitive tests) was modeled as a positive control.

**Results:**

Participant ages ranged from 67‐94 years, 60% were female and 36% identified as Black race (Table 1). As expected, audiometric hearing was not correlated with AD‐specific biomarkers (Ab42/40 ratio, *p*‐tau181). However, weak correlations were observed for markers more reflective of general neurodegeneration (NfL and GFAP) (Figure) and hearing loss was positively associated with NfL in adjusted linear regression models, with each 10 dB increase (worse hearing) associated with 0.079 (95% confidence interval [CI] 0.007, 0.150) higher log NfL (Table 2).

**Conclusions:**

Poorer audiometric hearing in older adults is associated with plasma biomarkers of neurodegeneration, specifically NfL, but is not associated with AD‐specific markers (amyloid and tau), suggesting that pathways linking hearing and ADRD are independent of these pathognomonic Alzheimer's‐related brain changes.